# The Effect of Art Expertise on Eye Fixation-Related Potentials During Aesthetic Judgment Task in Focal and Ambient Modes

**DOI:** 10.3389/fpsyg.2018.01972

**Published:** 2018-10-16

**Authors:** Agnieszka Fudali-Czyż, Piotr Francuz, Paweł Augustynowicz

**Affiliations:** Department of Experimental Psychology, The John Paul II Catholic University of Lublin, Lublin, Poland

**Keywords:** aesthetic judgment, art expertise, eye fixation-related potentials, focal mode, ambient mode, lambda response, N1, P2

## Abstract

This study aimed to determine the effect of expertise on the eye fixation-related potentials (EFRPs) during the aesthetic evaluation of images, independently in focal and ambient modes of visual processing. Focal and ambient modes were identified by averaging EFRP waveforms about the beginning of long eye fixations followed by short saccades and short fixations followed by long saccades, respectively. Thirty experts with formal training in visual arts and thirty-two non-experts freely viewed 150 figurative paintings presented for 20 s, each. After viewing the painting, the participant answered the question: “Is this painting beautiful?” Differences were found between the group of experts and non-experts due to the amplitude of EFRPs but only in focal mode, which is related to top-down, focused attention on the objects. Long fixations of experts had a higher amplitude of the parietal P2 recorded from right site than non-experts. In the group of experts, the frontal P2 was higher for long fixations on not beautiful paintings in comparison to long fixation on beautiful paintings. Moreover, in focal mode, there were higher occipital lambda response and N1-P2 complex for not beautiful than beautiful paintings. These results are discussed in the light of the results of studies on the effect of visual art expertise on event-related potentials (ERPs), ERP studies during aesthetic judgment task, and the knowledge of different modes of visual processing and EFRPs.

## Introduction

Experts in the field of art differ from non-experts in aesthetic preferences (Shimamura and Palmer, 2012) and characteristics of eye movement when viewing works of art (e.g., Nodine et al., 1993; Zangemeister et al., 1995; Francuz et al., 2018). However, it is still unclear whether visual art expertise impacts brain responses while viewing paintings to assess them aesthetically (i.e., before overt aesthetic decision). There were relatively few attempts to answer this question using event-related potentials (ERPs; ERP method see Luck, 2012) (e.g., Pang et al., 2013; Else et al., 2015) and eye movements were not controlled in these experiments. Recording synchronized ERPs, and eye tracking data is possible using eye fixation-related potential (EFRP) method (see Baccino, 2011; Nikolaev et al., 2014). EFRP enables analysis of brain activity during eye fixation on a specific part of the visual scene (Nikolaev et al., 2014). This method goes far beyond the traditional ERP approach giving the opportunity to analyze the way the visual scene is processed in different “fixation-saccade” sequences (Nikolaev et al., 2016) that are related to different modes of visual processing during free viewing (Velichkovsky et al., 2002, 2005; Tatler and Vincent, 2008; Pannasch and Velichkovsky, 2009). However, there are no EFRP studies on the effect of visual art expertise during aesthetic judgment task. Therefore, the goal of the present study was to investigate visual art expertise impact on EFRPs during aesthetic evaluation task, independently in different modes of visual processing.

EFRPs related to eye movements with different parameters differ in amplitude and latency (Graupner et al., 2011; Nikolaev et al., 2016). In turn, various combinations of the duration of fixation and saccade amplitude may indicate different ways of processing the visual scene (Velichkovsky et al., 2002, 2005; Tatler and Vincent, 2008; Pannasch and Velichkovsky, 2009). Short eye fixations combined with long subsequent saccades are indicators of the preattentive process of exploring the spatial organization of the visual scene in “ambient mode.” In turn, long eye fixations and subsequent short saccades are associated with focused attention on the object in “focal mode” of visual processing. There is evidence that the higher informativeness of objects or regions in an image, the more the focal mode of processing is engaged. For example, studies on driver’s reactions to hazardous driving situations showed that the appearance of an immediate hazard (e.g., red light) is related with the higher number of longer fixations (>601 ms) and reduced incidence of shorter fixations (90–300 ms) (Velichkovsky et al., 2002). On the contrary, the lower informativeness of individual elements of the visual scene (e.g., because of scrambling the picture content) is related to shortening of next fixations along with increasing of saccadic amplitudes (Foulsham et al., 2011).

The ambient mode is considering to be related to bottom-up processing and the focal mode, to top-down processing. In the bottom-up mode, information selection is depended on the properties of the image. In the top-down mode, information selection is under supervision according to the observer’s goals and knowledge, so that is associated strongly with the activation of working memory (Helo et al., 2014). In general, it is considered that the top-down attention control is more expressed in the case of experts than in non-experts (Hershler and Hochstein, 2009). We predicted more distinguishable EFRP responses during aesthetic judgment task, especially in experts than non-experts group, in the focal mode than in the ambient mode of visual processing, since the focal mode is associated with focused attention on the object (Velichkovsky et al., 2002, 2005; Tatler and Vincent, 2008; Pannasch and Velichkovsky, 2009) and top-down attention control (Helo et al., 2014).

The EFRP hypotheses in our study were based on the ERP research results (Wang et al., 2012; Noguchi and Murota, 2013; Pang et al., 2013; Else et al., 2015; Li et al., 2015). The EFRPs are treated like ERPs equivalents due to the topography and time of occurrence and because they are modulated depending on the cognitive task, such as object recognition or processing of the emotional content of the visual scene (Kazai and Yagi, 2003; Hutzler et al., 2007; Dandekar et al., 2012; Simola et al., 2015). For example, EFRP P2 (Simola et al., 2015) and ERP P2 (Herbert et al., 2006) were shown that could be modulated by the emotional processing of visual scene elements.

Regarding ERP studies on neural correlates of the effect of expertise in visual arts, Pang et al. (2013) showed that art experts’ neural activity is characterized by the lower P3b and LPC amplitudes recorded at the parietal leads, than non-experts, which is interpreted in the light of the neural efficiency hypothesis. On the other hand, Else et al. (2015) showed the enhanced amplitude of fronto-central N1 and the P2 component (recorded at occipital, parietal, and central sites) in artists compared to non-artists while viewing paintings. Based on Else et al. (2015) results, we expected a higher amplitude of parietal P2 in experts than non-experts, in focal than the ambient mode.

In studies on ERP responses during aesthetic judgment task, that did not consider the expertise factor, there is growing evidence that component P2 is a neural correlate of early aesthetic processing of visual scene (Wang et al., 2012; Noguchi and Murota, 2013; Li et al., 2015). Li et al. (2015) reported higher amplitude of fronto-central P2 for “not beautiful” Chinese typefaces compared to all Chinese characters and Wang et al. (2012) found a similar effect in case of “not beautiful” and “beautiful” pendants at the fronto-centro-parietal P2. They interpreted the “frontal” P2 effect as an expression of attentional bias caused by emotion arousal at the early stage of aesthetic evaluation, especially of less beautiful pictures. The opposite aesthetic effect seems to be true for the P2 component recorded mainly at parietal scalp area. Higher parietal P2 amplitude was found during the aesthetic evaluation of sculptures presented as original rather than counterfeits (Noguchi and Murota, 2013). The “parietal” P2 effect is considered to be related to a memory process in the aesthetic preference task. We predicted that there would be a higher amplitude of the parietal P2 while viewing paintings that are beautiful than not beautiful (Noguchi and Murota, 2013) and the opposite effect (not beautiful > beautiful) of the frontal P2 (Wang et al., 2012; Li et al., 2015).

Additionally, we analyzed not only the frontal and parietal P2 EFRPs recorded during eye fixations, separately in ambient and focal modes, but also another two occipital EFRPs – the P1 (lambda response, see Kazai and Yagi, 2003), and the N1-P2 complex (see Baccino, 2011). The higher amplitude of the occipital lambda response (Takeda et al., 2001; Yagi et al., 2010; Fudali-Czyż et al., 2014) and the occipital (Simola et al., 2009) or the occipito-parietal (Fischer et al., 2013) N1-P2 complex, the higher level of focused attention to objects on which the eye is fixed. Therefore, we have expected significant effects at the occipital lambda response (P1) and the N1-P2 complex time windows, especially in the focal mode of visual processing.

Previous research has provided evidence for the existence of the right-hemispheric dominance in the aesthetic preference task (Höfel and Jacobsen, 2007; Calvo-Merino et al., 2008; Cela-Conde et al., 2009; Noguchi and Murota, 2013). In ERP studies on an aesthetic evaluation, it was found a significant main effect of electrodes (P4 > P3, see Noguchi and Murota, 2013; C4 > C3, see Höfel and Jacobsen, 2007). Other studies also found the right-hemispheric dominance of the parietal cortex (Cela-Conde et al., 2009) or premotor cortex (Calvo-Merino et al., 2008) in the aesthetic evaluation of images or filmed dance movements. Also, Bromberger et al. (2011) found that patients with right hemisphere damage had significant difficulties evaluating most of the descriptive attributes of visual art compared to healthy subjects. So, we also expected right-lateralization of EFRP responses in the aesthetic evaluation task.

## Materials and Methods

### Participants

Thirty experts in the art (8 men, 22 women) and 32 non-experts (14 men, 18 women) took part in the study. The average age for experts was 24.4 years (*SD* = 1.7 years), and for non-experts 23.3 years (*SD* = 2.3 years). The experts were selected based on an objective criterion of education in the field of art (art history, painting, or graphic art). The group of non-experts included students of social sciences (psychology, pedagogy, sociology, and economics). They had normal or normalized eyesight and no history of neurological diseases. They were paid approximately US$ 15 for their participation in the study. The study was carried out by the recommendations of the Ethics Committee (Institute of Psychology at the John Paul II Catholic University of Lublin, Poland) with written consent from all participants.

### Stimuli

From a collection of 422 figurative paintings, six competent judges chose 309 with medium complexity and high-quality reproduction. The complexity of the image was defined by the number of presented objects, especially people, and the number of brightness and color contrasts that affect the potential bottom-up saliency effects. According to the first criterion, paintings, where there was only one person or one principal object in the foreground (e.g., a portrait or a jug with flowers) and more than five objects (e.g., a group of people), were rejected. The images in which the presented objects were strongly contrasting due to their brightness (e.g., in some Rembrandt or Goya paintings) or color (e.g., in some Matisse or van Gogh paintings) were also rejected. On the other hand, the criterion for high-quality image reproduction was purely technical. Images downloaded from the Internet have different resolutions, but for experimental purposes, all paintings have been standardized to the same height. Therefore, all those images which had lost their clarity due to the enlargement were rejected. Also rejected were images that were incomplete (e.g., cut off on one side) or they had distorted colors. After this selection, 26 non-experts (15 women, 11 men; age 23.3 years on average, *SD* = 2.2 years) rated the paintings as beautiful or not beautiful. From those, 150 paintings assessed as moderately beautiful (*M* = 30.67, *SD* = 5.21) were selected; those rated highly (*M* = 47.15, *SD* = 5.49) and low (*M* = 17.16, *SD* = 3.62) in terms of aesthetics were rejected.

Paintings were presented on a 24 ” (1920 × 1200 px) monitor placed 80 cm away from the participant in a dimly lit room on a gray background with a height of 1000 px (the vertical viewing angle for each one was fixed at 19.16 deg). Due to the specific character of stimuli, it was not possible to maintain a constant aspect ratio for all paintings, so as not to degrade their composition. The average horizontal viewing angle was 19.13 deg (*SD* = 5.67, max = 34.12).

### Apparatus

In the EFRP investigation, we used an eye tracker (SMI iView X Hi-Speed) synchronized with an electroencephalograph with a high-input impedance amplifier (200 MOhms, EGI Inc., Model: GES 300), using an active electrode system (Brain Products 64-channel actiCAP) referenced to averaged mastoids. Net Station 4.4 was used in EEG registration (sampling rate 500 Hz; electrodes impedance was kept below 5 kOhm) and iViewX 2.8 for eye movement recordings (registration of the position of the right (dominant) eye with a sampling rate of 500 Hz). Eye calibration (13-point) was carried out at the beginning of the test and every 15 successive experimental trials (validation: max eye position error accepted -1° or less). Eye tracking and EEG data streams were synchronized using common trigger pulses sent from experimental control software via parallel port. Sync error reported by EYE-EEG toolbox^1^ used for syncing these data streams did not exceed + /- 1 data sample (2 ms at a rate of 500 Hz) (Dimigen et al., 2011).

### Procedure

The trial began with the appearance of a central fixation point on the screen for 1.250 ms. Participants were asked to fix their gaze on it. Then the painting was displayed for 20 s. After each painting was shown, participants answered the question: “Is this painting beautiful?” by pressing the Yes or No button on the response pad.

### Data Analysis

Co-registered EEG and eye movement data were analyzed in MATLAB with EEGLAB/ERPLAB toolbox and EYE-EEG extension. A band pass of 0.1–40 Hz was used for EEG signal filtering. Saccades and fixations were detected using an adapted version of the velocity-based Engbert and Mergenthaler’s algorithm (Engbert and Mergenthaler, 2006) using the parameters described in Kamienkowski et al. (2012a,b): velocity threshold for saccade detection 6 *SD*, minimum saccade duration 4 samples (× 2 ms) = 8 ms, raw data smoothed to suppress noise, adaptive velocity threshold, 50 ms minimum allowed fixation duration between two saccades. We kept only the largest saccades of each temporal cluster of saccades; only saccades larger than 1° were kept for further analyses (Engbert and Kliegl, 2003). We removed fragments of EEG recordings contaminated by eye blinks with extra + /- 50 ms of data before and after eye blinks. Before segmentation, EEG data was cleaned using artifact subspace reconstruction procedure (Mullen et al., 2015).

**FIGURE 1:**
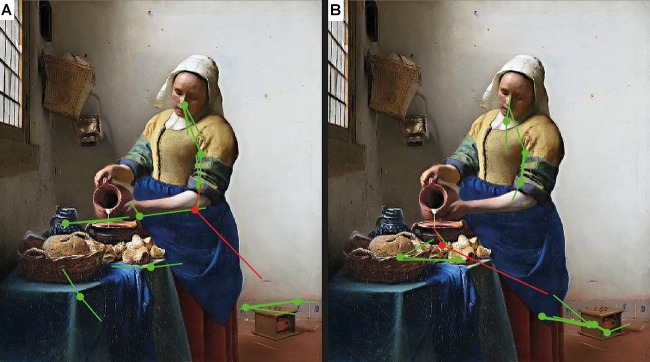
Example of focal and ambient mode of visual processing from one of 32 non-experts **(A)** and one of 30 experts **(B)** recorded while viewing one of the 150 paintings included in the experiment (Johannes Vermeer, *Woman with a Water Jug*, 1660–1662, oil on canvas, 45.7 × 40.6 cm, Metropolitan Museum of Art, New York City, NY, United States). In green are marked long fixations (>180 ms) followed by short saccades (≤4°); in red are marked short fixations (≤180 ms) followed by long saccades (>4°).

**FIGURE 2:**
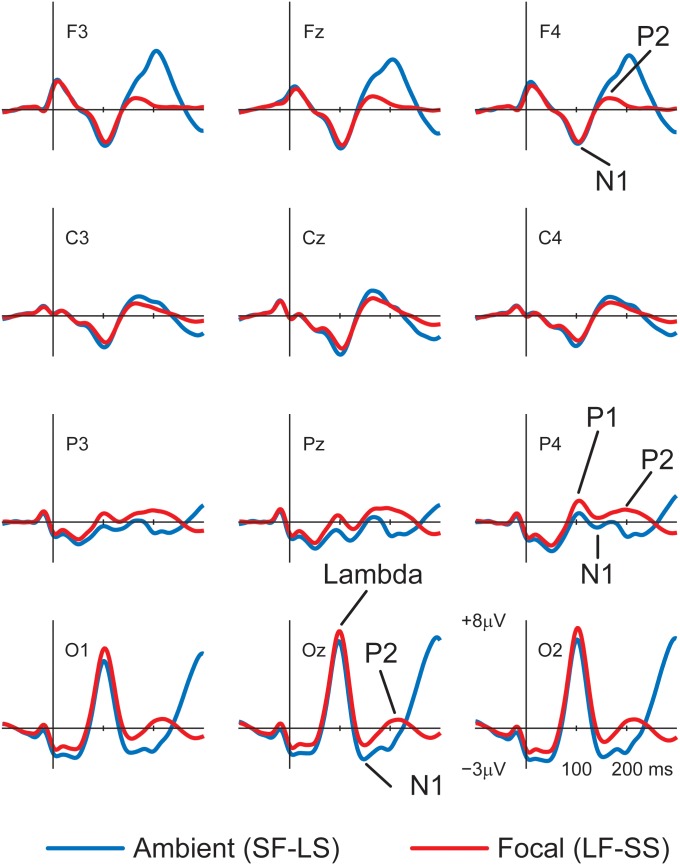
Grand average EFRP waveforms from 62 participants generated separately for fixations belonging to focal (see red line) and ambient (see blue line) modes.

We were interested in the analysis of the effects of expertise on the amplitude of individual EFRPs during the aesthetic task, separately for the eye movement sequence indicating focal and ambient mode (see **Figure 1**). We have decided to carry out separate analyses for two categories of eye movements (ambient and focal mode) because according to current knowledge, and our preliminary data analysis (see **Figure 2**), eye movements parameters have a significant impact on the shape and the amplitude of the EFRP recording (Nikolaev et al., 2016). Because it was shown that the amplitude of fixation onset-related EFRPs is particularly sensitive to the amplitude of the preceding saccade (Figure 11 in Nikolaev et al., 2016, p. 71–72), we limited the analyses of fixation-saccade sequences to those which were preceded by a short saccade.

Before data analyses, all valid fixations (>100 ms; Pannasch and Velichkovsky, 2009) were classified according to their durations and amplitudes of following saccades. The criterion for fixations durations and subsequent saccades amplitudes classification (short vs. long) was set at 180 ms (Pannasch and Velichkovsky, 2009) and 4 deg of visual angle (Wyszecki and Stiles, 1982), respectively (see **Figure 1**). As stated by von Wartburg et al. (2007), the mean saccade length could be predicted by the image size operationalized by a maximal extent in either horizontal or vertical direction, in deg. The mean size of our stimuli was 21.70 (*SD* = 3.53) deg. Therefore, the mean saccade length predicted by image size was 4.38 deg, which was close to the threshold used in the present study (4 deg). The mean saccade amplitude computed for our dataset was 4.58 deg.

The mean fixation duration of ambient and focal sequences were 302.14 ms (*SD* = 7.08 ms) and 684.50 ms (*SD* = 70.35 ms), respectively. We adjusted the width of EEG segments to the average duration of short fixations. EEG data were segmented between -100 ms and 300 ms aligned to fixation onset. We used a simple voltage threshold of + /-70 uV to remove segments with remaining EEG artifacts. The mean number and standard deviation of fixations in focal and ambient sequences (with short preceding saccades) entering the EFRP analyses across participants and experimental conditions (expertise and aesthetic judgment) are shown in **Table 1**. Every eye fixation of each participant was categorized as “eye fixation on a beautiful/not beautiful painting” after evaluation given painting as beautiful or not beautiful. The mean number of fixations in the EFRP analyses differed between experimental conditions. This is natural considering the task, which allowed participants to look at different parts of the paintings freely.

**Table 1:** Mean number (*SD*) of fixations in two different fixation – saccade sequences entering the EFRP analyses across participants and experimental conditions.

Fixation – saccade sequences (modes)	Aesthetic judgments	Experts	Non-experts
Focal	Beautiful	485.27	351.78
		(33.59)	(32.52)
	Not beautiful	315.57	370.38
		(28.28)	(27.38)
Ambient	Beautiful	102.33	76.84
		(8.01)	(7.76)
	Not beautiful	78.83	87.06
		(7.82)	(7.57)

Before plotting and exporting data for statistical analysis, the segments were baseline corrected. The baseline for each channel was defined between -100 ms and -50 ms before the onset of the current fixation. We selected the baseline considering that preceding saccades were shorter than 50 ms (*M* = 20.25, *SD* = 3.27) because we rejected eye movements sequences with long preceding saccades. This way, we could assure that the impact of saccadic activity was similar for all conditions (cf. Kamienkowski et al., 2012a). There were no differences between experimental conditions and between expertise groups at a mean EFRP amplitude in the time window from -50 to 0 ms before fixation onset (*ps* > 0.250).

In grand average EFRP waveforms belonging to both focal and ambient modes, the following components were visible: the lambda response, the N1-P2 complex at occipital scalp areas, the P1 at parietal scalp areas, and the N1 and the P2 at parieto-centro-frontal scalp areas (cf. Kazai and Yagi, 2003; Hutzler et al., 2007; Graupner et al., 2011; Dandekar et al., 2012; Fischer et al., 2013; Simola et al., 2015 Nikolaev et al., 2016) (see **Figure 2**).

Further visual inspection of grand average EFRP waveforms and topographies suggested that several EFRPs were sensitive to expertise and/or aesthetic judgment: the occipital lambda response (78–118 ms), the occipital N1-P2 complex (135–235 ms), parietal P2 [focal mode: 160–230 ms; ambient mode: 140–190 ms], and frontal P2 [focal mode: 140–190 ms; ambient mode: 160–230 ms). For the lambda response and the N1-P2 complex, we restricted our analyses to three occipital channels: O1, Oz, and O2 (cf. lambda response: Kazai and Yagi, 2003; Fudali-Czyż et al., 2014; the N1-P2 complex: Baccino, 2011; the occipital P2: Simola et al., 2009; Graupner et al., 2011). For the parietal P2 component, we focused on three parietal electrodes: P3, Pz, and P4 (cf. Noguchi and Murota, 2013). For the frontal P2 component, we focused on three frontal channels: F3, Fz, and F4 (cf. Wang et al., 2012; Li et al., 2015; Simola et al., 2015).

We conducted eight repeated measure ANOVAs with expertise (experts, non-experts), aesthetic judgment (beautiful, not beautiful), and laterality (left/middle/right) as factors, for the lambda response, N1-P2 complex, parietal P2, and frontal P2 time windows, separately for the condition of focal and ambient subsequence. The effects of laterality are reported only when they interact with any of the other two factors. A Greenhouse-Geisser correction was applied when appropriate.

## Results

### Lambda Response (P1) Time Window (78–118 ms)

**FIGURE 3:**
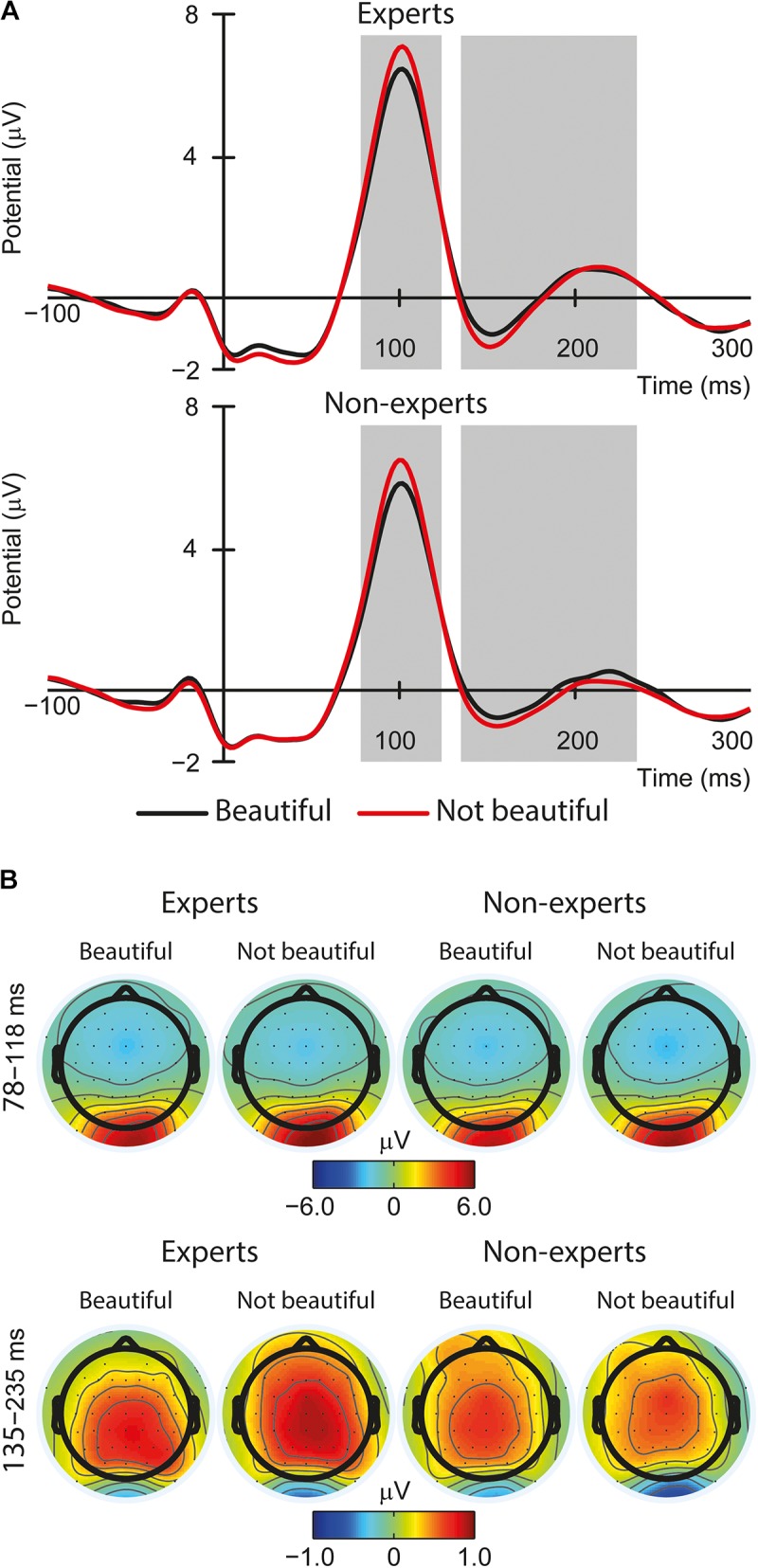
**(A)** The effect of the aesthetic judgment (beautiful vs. not beautiful) on the lambda response and the N1-P2 complex averaged amplitude from occipital electrodes (O1, Oz, and O2) in the focal mode (EFRPs time windows are indicated by the gray fields). **(B)** Topographic maps of fixation onset – related brain activity in two-time windows (focal mode): 78–118 ms (lambda response) and 135–235 ms (N1 – P2 complex).

For focal mode, the main effect of aesthetic judgment appeared to be significant [*F*(1,60) = 25.92, *p* < 0.001, part. η^2^ = 0.30; see **Figures 3A,B**]. There was a higher lambda response in the case of not beautiful paintings (*M* = 4.78, *SE* = 0.27) compared to beautiful ones (*M* = 4.33, *SE* = 0.27). There were no other significant effects (*Fs* < 0.90, *ps* > 0.350). For ambient mode, there were no significant effects (*Fs* < 1.26, *ps* > 0.262).

### N1-P2 Complex Time Window (135–235 ms)

For focal mode, we found a significant effect of aesthetic judgment [*F*(1,60) = 12.19, *p* = 0.001, *part.*η^2^ = 0.17; see **Figures 3A,B**]. There was a lower mean amplitude of the N1-P2 complex for not beautiful paintings (*M* = -0.60, *SE* = 0.18) compared to beautiful ones (*M* = -0.36, *SE* = 0.16). There were no other significant effects (*Fs* < 0.16, *ps* > 0.698). For ambient mode, there were no significant effects (*Fs* < 3.37, *ps* > 0.071).

### Parietal P2 Time Windows (Focal Mode: 160–230 ms; Ambient Mode: 140–190 ms)

**FIGURE 4:**
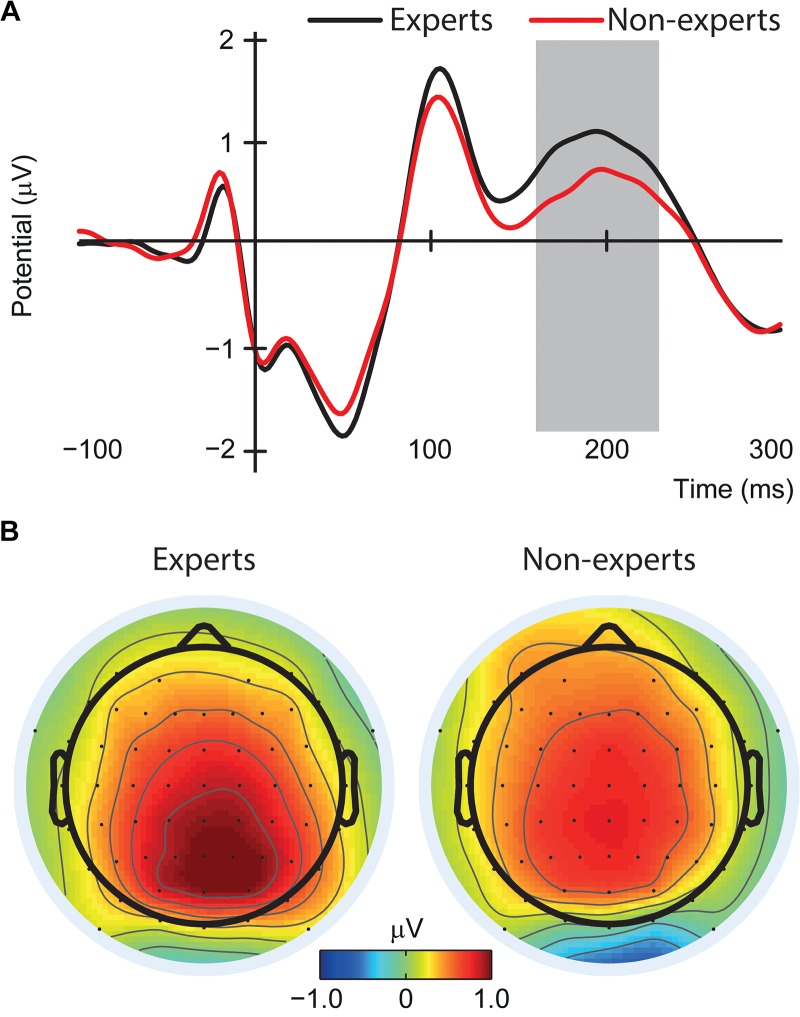
**(A)** The effect of expertise on the P2 amplitude recorded from the right parietal electrode (P4) in the focal mode (collapses for eye fixations on beautiful and not beautiful paintings) – the parietal P2 time window is indicated by the gray field; **(B)** Topographic maps of fixation onset – related brain activity in 160–230 ms time window (focal mode).

For focal mode, the interaction effect of expertise and laterality appeared to be significant [*F*(2,120) = 4.92, *p* = 0.011, *part.*η^2^ = 0.14]. There was higher mean amplitude of the P2 recorded from the right parietal scalp area (P4) in the expert group (*M* = 0.93, *SE* = 0.10) in comparison to the non-expert group (*M* = 0.53, *SE* = 0.10) (*p* = 0.005). The **Figures 4A,B** present the effect of expertise on the parietal P2 amplitude recorded from right parietal electrode (P4) in the focal mode (collapses for eye fixations on beautiful and not beautiful paintings) and topographic maps of parietal P2 in 160–230 ms time window. There were no differences in recording from left (P3) and middle (Pz) parietal electrodes (*ps* > 0.105). There were no other significant effects (*Fs* < 3.83, *ps* > 0.055). For ambient mode, there were no significant effects (*Fs* < 2.15, *ps* > 0.124).

### Frontal P2 Time Windows (Focal Mode: 140–190 ms; Ambient Mode: 160–230 ms)

**FIGURE 5:**
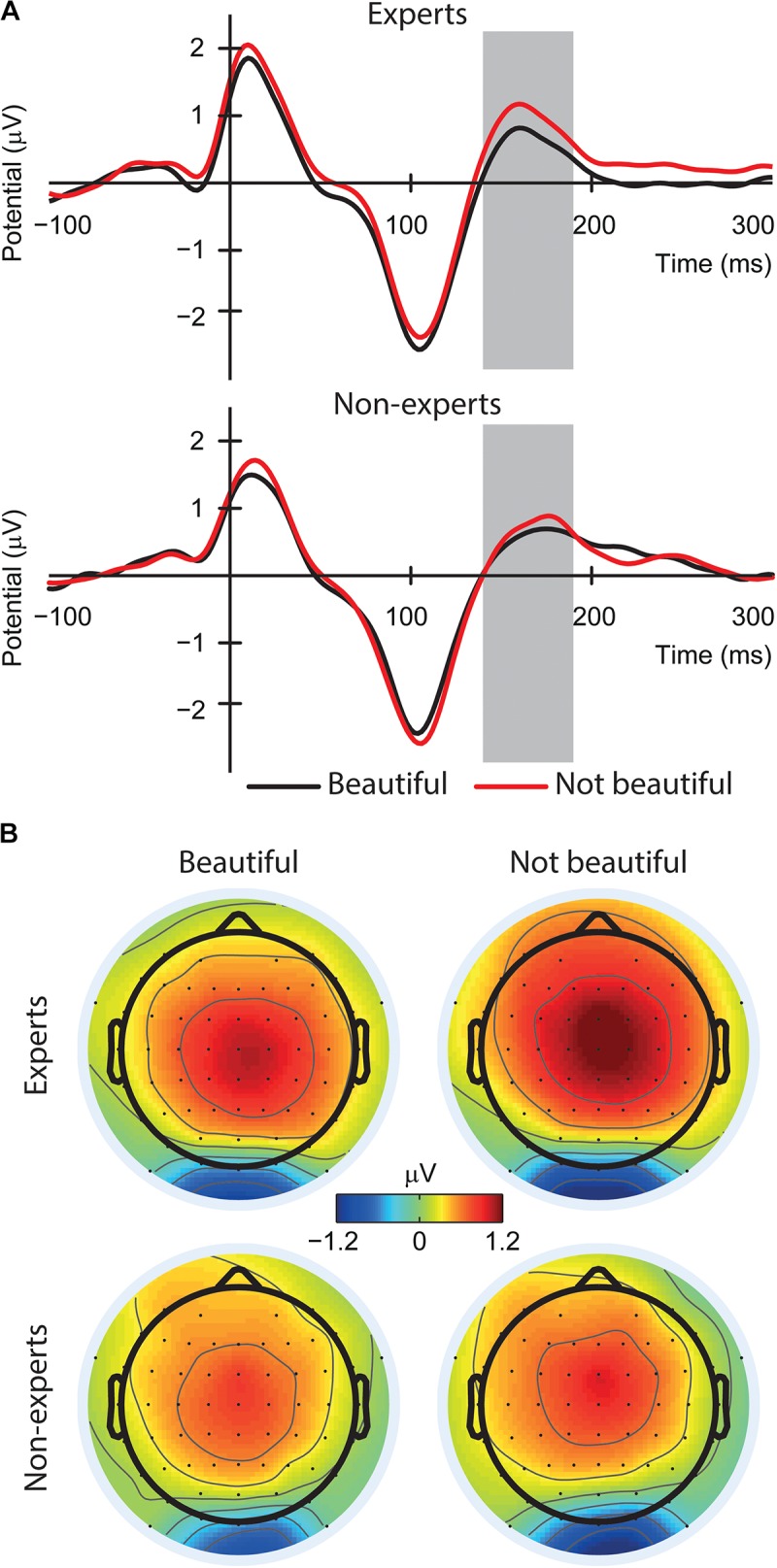
**(A)** The interaction effect of expertise and aesthetic judgment on the frontal P2 amplitude (averaged from F3, Fz, F4 electrodes) in the focal mode (the frontal P2 time window is indicated by the gray field). **(B)** Topographic maps of fixation onset – related brain activity in the 140–190 ms time window (focal mode).

For focal mode, there was significant interaction of expertise and aesthetic judgment [*F*(1,60) = 4.66, *p* = 0.035, *part.*η^2^ = 0.07, see **Figures 5A,B**]. In the expert group, there was higher frontal P2 amplitude in case of not beautiful (*M* = 0.61, *SE =* 0.12) compared to beautiful paintings (*M* = 0.32, *SE* = 0.12) (*p* = 0.008). There was no aesthetic judgment effect in the non-expert group between not beautiful (*M* = 0.35, *SE* = 0.13) and beautiful paintings (*M* = 0.37, *SE* = 0.12) (*p* = 0.801). There were no other significant effects (*Fs* < 0.90, *ps* > 0.350). For ambient mode, there were no significant effects of expertise and aesthetic judgment variables (*Fs* < 1.00, *ps* > 0.325).

## Discussion

The present EFRP study aimed to examine the impact of visual art expertise on the EFRPs during the aesthetic judgment task, independently for focal and ambient modes of visual scenes processing. We expected a more significant effect of the expertise variables on the EFRPs in a focal rather than an ambient mode during aesthetic judgment task. The results of the current study confirmed this assumption.

It turned out that only focal mode differentiated the experimental conditions regarding four EFRP time windows: 78–118 ms (lambda response), 135–235 ms (N1-P2 complex), 160–230 ms (parietal P2), and 140–190 ms (frontal P2). In case of the ambient mode of visual processing, there were no significant effects at any of the analyzed EFRP time windows. It is believed that focal mode of visual processing in combination with long fixations and short saccades served visual object identification (Velichkovsky et al., 2002, 2005; Tatler and Vincent, 2008; Pannasch and Velichkovsky, 2009) and is strongly related with top-down visual processing (Helo et al., 2014). Therefore, it can be assumed that focal fixations are not accidental and serve observers internal goals that are task-relevant, and therefore also those fixations are related to the brain activity pattern that is characteristic for a given task.

Only in the focal mode, fixations of experts in comparison to non-experts were characterized as expected by a higher amplitude of the parietal P2 recorded from right electrodes. This effect is in line with ERP study results showing – on the one hand – the higher amplitude of cortical brain activity in experts than in non-experts in aesthetic evaluation tasks (Else et al., 2015), and – on the other hand – the existence of the right-hemispheric dominance in the aesthetic preference task (Höfel and Jacobsen, 2007; Calvo-Merino et al., 2008; Cela-Conde et al., 2009; Noguchi and Murota, 2013). The parietal P2 amplitude is considered to be related to the memory bias (Noguchi and Murota, 2013), which is one of the top-down factors differentiating experts and non-experts (Harel, 2016). Previous ERP studies showed that not only the P2 with the dominant parietal scalp distribution but also the P2 with more frontal topography might be considered as a neural correlate of early aesthetic processing of visual scene (Wang et al., 2012; Li et al., 2015). While, there was no main effect of aesthetic evaluation in case of the parietal P2 (cf. Noguchi and Murota, 2013), the results confirmed the hypothesis of a higher amplitude of the frontal P2 for fixations on not beautiful paintings in comparison to beautiful ones (cf. Wang et al., 2012; Li et al., 2015), but only in a group of experts in focal mode. Previous studies have shown that frontal P2 amplitude increases not only for both positive and negative stimuli when compared with the neutral stimuli (Herbert et al., 2006) but also for not beautiful pictures in comparison to beautiful ones (Wang et al., 2012; Li et al., 2015). The “frontal” P2 effect is interpreted as an expression of attentional bias caused by emotion arousal at the early stage of aesthetic evaluation, especially of less beautiful pictures (Wang et al., 2012; Li et al., 2015). It is known that emotions are an essential element of aesthetic preference (Chatterjee, 2004).

There was no main effect of the “frontal” P2 modulation in the focal mode during aesthetic judgment task even though there was the higher amplitude of occipital lambda response and the N1-P2 complex during long fixations followed by short saccades registered on paintings rated as not beautiful compared to beautiful ones. It may have resulted from the primary sensory discrimination process within the focus of attention which was stronger to stimuli later assessed as not beautiful than beautiful (cf. Vogel and Luck, 2000). Occipital EFRPs, the lambda response (Takeda et al., 2001; Yagi et al., 2010; Fudali-Czyż et al., 2014) and the N1-P2 complex (Simola et al., 2009; Baccino, 2011) are treated as cerebral correlates of focused attention.

Fischer et al. (2013) suggested that the increase of the N1-P2 complex mean amplitude (the N1 decrease and the P2 increase) is related to a general bias toward top-down modulations across inspection time. They observed a simultaneous increase in fixation duration and the N1-P2 complex amplitude as a function of the viewing time of presented paintings. In the present study, at the similar time window as the occipital N1-P2 complex (135–235 ms) - the frontal P2 effect (140–190 ms) occurred, but only in an expert group. It seems that only in the expert group, eye fixations on not beautiful paintings in a focal mode co-occurred with both the higher focused attention and greater emotional arousal than eye fixations on beautiful paintings.

The expertise effect was not found in EFRP recordings from occipital electrodes but instead was found at the fronto-parietal scalp areas in the focal mode of visual processing. There is evidence that the fronto-parietal feedback system takes part in the programming of the spatial exploration of the scene and occipital and temporal cortex with more stimuli-driven analysis of features of visual input (Corbetta et al., 2008). Therefore, expert knowledge may lead to the predominance of top-down over bottom-up processes during the aesthetic evaluation of visual art, and this is manifested by the modulation of the activity of the fronto-parietal feedback system in the focal mode. The results of neuroimaging studies reveal that visual expertise is achieved by the deployment of top-down control mechanisms, while the level of knowledge is related to the enhancement in the large-scale top-down attentional networks (Harel et al., 2013; Harel, 2016). It seems that non-perceptual factors play a critical role in distinguishing experts from non-experts because both groups differed regarding visual task performance even though they received the same set of stimuli (Harel et al., 2013). For example, in one study, bird and car experts searched for face, car, or bird photographs. The car experts were faster and more accurate when looking for cars than when looking for birds. The bird experts were also significantly faster when looking for birds than when looking for a car (Hershler and Hochstein, 2009). In another study, it was shown that non-experts more often than experts do not notice changes in the details of visual scenes, despite looking directly at the area of change (Fudali-Czyż et al., 2014).

To the best of our knowledge, our EFRP research on the cortical brain activity related to different modes of aesthetic visual processing in a group of experts and non-experts is to this date unique in two respects. First, so far there has been no EFRP research in the visual art domain with explicit aesthetic evaluation with expertise factor. Second, we conducted our EFRP analyses separately for two kinds of visual processing modes (focal and ambient), taking into account two different eye fixation-saccade sequences, respectively. We based our hypotheses as analogous to the ERP results on visual art expertise and the aesthetic evaluation of visual stimuli and by referring to results of EFRP studies and eye-tracking research conducted in research areas other than the visual art aesthetic processing domain. We showed that the effect of the visual art expertise on the amplitude of EFRPs during aesthetic judgment task reveals itself in the focal processing mode (i.e., long fixations and shortly followed saccades). We also demonstrated there was no modulation of EFRPs in the ambient mode with short fixations followed by long saccades. Further studies are needed to test the relation among brain activity, eye movement context, and aesthetic visual processing in art experts and non-experts. It is especially important to think about designing research with aesthetic judgment tasks that have higher ecological validity, enabling easier transfer to real experience in the art gallery (cf. Carbon, 2017).

## Ethics Statement

We confirm that APA ethical standards were followed in the conduct of the study. This study was carried out in accordance with the recommendations of the Ethics Committee (Institute of Psychology at the John Paul II Catholic University of Lublin, Poland) with written consent from all subjects. All subject gave informed consent in accordance with the Declaration of Helsinki. This study was approved by Research Ethics Committee of the Institute of Psychology of The John Paul II Catholic University of Lublin.

## Author Contributions

AF-C, PF, and PA provided substantial contributions to the conception and design of the work and acquisition, analysis, or interpretation of data for the work. AF-C and PF drafted and revised the work critically for important intellectual content, approved the final version to be published, and agreed to be accountable for all aspects of the work in ensuring that questions related to the accuracy or integrity of any part of the work are appropriately investigated and resolved.

## Conflict of Interest Statement

The authors declare that the research was conducted in the absence of any commercial or financial relationships that could be construed as a potential conflict of interest.

## Footnotes

1 http://www2.hu-berlin.de/eyetracking-eeg
